# Association of handgrip strength with the plasma metabolomic profile: secondary analysis of a protein intervention study

**DOI:** 10.1007/s11306-026-02496-4

**Published:** 2026-06-30

**Authors:** Tilde Martinsen, Janine Wirth, Lorraine Brennan

**Affiliations:** 1 UCD School of Agriculture and Food Science, University College Dublin, Dublin 4, Ireland; 2 Conway Institute, University College Dublin, Dublin 4, Ireland; 3 UCD School of Agriculture and Food Science, UCD Institute of Food and Health, Belfield, Dublin 4, Ireland

**Keywords:** Dietary protein quality, Metabolomics, Muscle strength, Plant-protein, Handgrip strength

## Abstract

**Introduction:**

There is interest in the potential impact of increased protein intake on muscle function in older adults. However, there is paucity of information on effects of different protein types.

**Objectives:**

To determine the impact of protein sources (plant vs dairy) on the plasma metabolomic profile, and to explore associations between metabolites and handgrip strength.

**Methods:**

In this secondary analysis of a 12-week double-blinded RCT, 171 healthy adults (≥50 years) were assigned to high plant protein (23 g/d), high dairy protein (22 g/d) or low protein (2 g/d) supplement. Plasma samples were collected pre- and post-intervention and analysed using a targeted LC-MS platform. Main and interaction effects were estimated using ANOVA-simultaneous component analysis (ASCA). Spearman correlation analysis evaluated associations between metabolites and handgrip strength, with false discovery rate correction (q < 0.05).

**Results:**

ASCA revealed no significant main effect of group (p = 0.23) or group × time interaction (p = 1.00), while time had a significant effect on the metabolomic profile (p < 0.05). After FDR correction, 26 metabolites were significantly correlated with baseline handgrip strength. The strongest positive correlations were observed for creatinine (r = 0.53), leucine (r = 0.38), proline (r = 0.35) and isoleucine (r = 0.33). Baseline levels of leucine, isoleucine, PCaeC322 and SMOHC222 were associated with positive changes in handgrip strength (p < 0.05).

**Conclusions:**

Metabolomic changes were not related to protein type. However, metabolite levels were correlated with handgrip strength outcomes, suggesting the necessity to evaluate the metabolic state of individuals prior to interventions.

**Trial registration:**

This study is registered on isrctn.com as ISRCTN16651067.

**Supplementary Information:**

The online version contains supplementary material available at https://doi.org/10.1007/s11306-026-02496-4.

## Introduction

Age-related decline in muscle strength is a multifactorial process, influenced by modifiable factors, such as low physical activity, inadequate protein intake and poor nutritional status (Denison et al., 2015). Proposed underlying biological drivers includes chronic low-grade inflammation, age-related endocrine alterations, increased oxidative stress and accumulation of advanced glycation end-products that adversely affect skeletal muscle structure and function (Butler-Browne et al., 2013; Dalle et al., 2017; Granic et al., 2023; Gupta & Kumar, 2022; Liang et al., 2022). However, the metabolic pathways linking these processes to age-related strength loss remain incompletely characterised.

In this context, metabolomic profiling provides a systems-level approach to identify biomarkers and complex changes in metabolic pathways relevant to skeletal muscle aging (Brennan & de Roos, 2023; Chen & Wu, 2024; Lin et al., 2024; Qiu et al., 2023). Accordingly, multiple metabolite classes, including amino acids, acylcarnitines, fatty acid amides and gut microbiota-derived metabolites, have been associated with muscle strength and early risk of sarcopenia (Guo et al., 2024; Kim et al., 2023). More recently, specific lysophosphatidylcholines have been identified as independent predictors of physical performance and mitochondrial oxidative capacity in older adults (Klingler et al., 2016; Semba et al., 2019).

Alongside these metabolic determinants, adequate dietary protein intake remains a key modifiable factor for muscle preservation, yet a substantial proportion of older adults fail to meet recommended intakes (Hengeveld et al., 2020). To counter age-related anabolic resistance and support maintenance of lean mass and function, expert groups recommend higher protein intakes of approximately 1.0–1.2 g/kg body weight/d for healthy older adults (Bauer et al., 2013; Paddon-Jones et al., 2015; Paddon-Jones & Leidy, 2014; Volkert et al., 2022). At the same time, dietary guidelines increasingly encourage a greater proportion of protein intake to come from plant-based sources (Willett et al., 2019). This shift is supported by prospective evidence linking higher plant-protein intake with lower all-cause and cardiovascular mortality risk, and by evidence that most plant-protein foods have substantially lower environmental impacts compared with animal-source protein (Naghshi et al., 2020; Willett et al., 2019). Nevertheless, animal-derived proteins are considered higher quality than plant protein sources due to a more favourable indispensable amino-acid profile and greater bioavailability (Gorissen & Witard, 2018; FAO, 2013), raising the possibility that protein source may differentially affect pathways relevant to muscle maintenance.

In observational studies of older adults, circulating branched-chain amino acids (BCAAs), including leucine, isoleucine and valine, have been associated with skeletal muscle mass (Lustgarten et al., 2014), and in some cohorts also been associated with measures of muscle strength and physical function, (e.g., handgrip strength and gait speed) (Ter Borg et al., 2019). However, higher baseline levels of BCAAs and aromatic amino acids have also been prospectively associated with increased risk of incident type 2 diabetes among initially normoglycemic individuals (Wang et al., 2011). Furthermore, sphingolipids, particularly ceramides are bioactive membrane lipids implicated in insulin resistance and in skeletal-muscle lipid signalling processes relevant to muscle ageing and contraction (Chavez & Summers, 2012; Tan-Chen et al., 2020), and circulating ceramide species have also been prospectively associated with incident type 2 diabetes (Fretts et al., 2020). However, their associations with muscle strength and function in aging remain poorly understood (Tan-Chen et al., 2020). Characterising metabolomic signatures in response to dietary protein sources of different quality, and relating these signatures to muscle strength, may clarify pathways underlying age-related decline in muscle strength and inform targeted nutritional strategies. Furthermore, it remains unclear whether plant- and dairy-derived proteins elicit distinct metabolomic responses and whether these profiles are associated with muscle function.

Therefore, in this secondary analysis of a 12-week randomised controlled trial, the objective was to examine the effects of plant- and dairy-derived protein sources on circulating metabolite profiles and to examine associations between metabolomic profiles and muscle function.

## Materials and methods

### Study design and participants

This was a secondary analysis of a double-blinded randomised controlled trial conducted in healthy adults aged 50 years and older (Wirth et al., 2024). A total of 171 eligible participants, 100 women and 71 men, were randomly assigned into one of three products, which were consumed daily for 12-weeks: plant-based protein shake (23 g. protein/d, *n* = 60), dairy-based protein shake (22 g. protein/d, *n* = 56) or low-protein shake (2 g. protein/d, *n* = 55). Participants were instructed to maintain their usual diet and physical activity in conjunction with the shake. Clinical and functional measures, including handgrip strength, anthropometry and fasting plasma were collected at baseline and after 12-week intervention. Randomisation was performed by a researcher external to the study using blocked randomisation (https://www.studyrandomizer.com/).

Participants were recruited from the general population via advertisements posted on social media, e-mail, and flyers. All subjects were adults aged ≥ 50 years, of general good health, and free from chronic or metabolic disease and other conditions that could alter energy metabolism or nutritional requirements. The sample size was calculated using G*Power software version 3.1, based on analyses of primary outcome (handgrip strength) and protein intake (Wirth et al., 2024). The study was conducted in a research suite at University College Dublin. The trial was carried out between October 2021 and January 2023 in accordance with the declaration of Helsinki, and all participants gave their written informed consent before study begin. The ethics review board of the University College Dublin (LS-21–61-Wirth-Brennan) approved all study procedures. The trial was registered at isrctn.com as ISRCTN16651067.

Data collection was performed following a 12-h overnight fast and occurred at baseline and at the end of the intervention. Briefly, fasting blood was collected into a lithium heparin vacutainer. The samples were centrifuged at 1800 g for 10 min at 4 °C, and stored at −80 °C until analysis.

Handgrip strength (primary outcomes) was assessed using a handheld dynamometer (Takei 5401, Japan). Participants stood upright with their arms down naturally at their sides. They were instructed to squeeze the handgrip dynamometer four times (twice per hand), with a 30-second rest between measurements. Following the manufacturer’s guidelines, the average of the highest values recorded for each hand (in kilograms) was used for analysis. If one arm was injured, the highest value from the healthy arm was used. Handgrip strength changes (ΔHGS) were calculated as the 12-weeks (postintervention) values minus the baseline values. Associations between baseline metabolites and ΔHGS were examined among participants. Because no significant group × time effects were observed, participants were pooled for the secondary analyses examining associations between circulating metabolites and handgrip strength.

### Metabolomic analysis

Plasma samples were analysed using the AbsoluteIDQ p180 kit for targeted metabolite analysis according to the manufacturers’ instructions and as previously described (Yin et al., 2022). Samples were analysed in a randomised order. A total of 10 µL of sample (plasma, Biocrates quality controls (QC), PBS, or calibration standards) was added into the 96-well plate and dried under nitrogen for 30 min at room temperature. Derivatization solution (5% phenyl isothiocyanate in ethanol/water/pyridine (ratio 1/1/1, v/v/v)) was added to each well and incubated for 25 min at room temperature. Drying was achieved using nitrogen for 60 min. Metabolites were then extracted with 300 µL 5 mM ammonium acetate in methanol and the plate was centrifugated at 500×*g* for 2 min. 150 µL of the eluate was diluted with 150 µL HPLC grade water for the LC-MS/MS analysis, and 50 µL of eluate was diluted with 450 µL mobile phase for flow injection analysis–tandem mass spectrometry (FIA-MS/MS) analysis.

The data was acquired on a Sciex ExionLC series UHPLC system coupled to a Sciex QTRAP 6500 + mass spectrometer. System suitability tests were performed before start of acquisition. Two testmix solutions provided by Biocrates were used to assess the LC-MS and FIA acquisition. For the LC part, the MS sensitivity, retention times and column condition were examined. For the FIA part, the condition of the system and the ion spray stability were assessed. Liquid chromatography was performed on a AbsoluteIDQ^®^ p180 Kit UHPLC column using acetonitrile and water with 0.2% formic acid as mobile phase. Amino acids and biogenic amines were quantified in positive mode. For the FIA-MS/MS analysis, data was acquired using methanol as the running solvent.

### Data processing

Data evaluation for the quantification of metabolites and quality assessment was performed with the MetIDQ™ software (Biocrates). Data quality was evaluated by checking the accuracy and reproducibility of QC samples included in the p180 assay. Furthermore, analysis of the pooled plasma QC revealed that 96% of the metabolites reported had a CV < 20%. Amino acids and biogenic amines were quantified based on internal standards and calibration curves consisting of seven calibration standards, while acylcarnitines, LPCs, PCs, SMs and hexose were evaluated semi-quantitatively by using 13 internal standards. Metabolite concentrations are reported in µM. Metabolites were removed from further analysis when the metabolite was found in concentrations below the limit of detection (LOD) in more than 75% of plasma samples.

### Statistical analysis

All statistical analyses were performed using R (version 4.4.0) and MetaboAnalyst (version 6.0). Data are presented as mean ± SD unless otherwise indicated. Spearman rank correlation was used to identify metabolites associated with handgrip strength outcomes. For each baseline correlation, unadjusted P values were calculated and corrected for multiple testing using the Benjamini-Hochberg false discovery rate (FDR). Statistical significance was defined as FDR-adjusted P values (q) < 0.05. ANOVA-simultaneous component analysis (ASCA) was performed to evaluate the main effects of time, group, and the time × group interaction on the metabolomic data. Prior to ASCA, hexose metabolites were excluded and data were unit-variance scaled. Statistical significance of ASCA effects was assessed using 1,000 permutation tests. For ASCA feature selection, an α threshold of 0.05 (SPE) and a leverage threshold of 0.9 were applied. Unless otherwise specified, tests were two-sided with *P* ≤ 0.05 considered statistically significant.

## Results

### Baseline characteristics and handgrip strength across groups

A total of 171 participants (mean age 59.2 ± 7.7 years) were included in this secondary analysis. Overall, 59% were female and the average BMI was 26.2 ± 4.9 kg/m². Baseline characteristics did not differ significantly across the three study groups with respect to age, sex, or BMI (all *P* > 0.3; Table S1). Handgrip strength was similar across all groups at baseline (27.9 ± 9.0 kg). Changes in handgrip strength over the 12-week intervention period did not differ significantly between the groups (Wirth et al., 2024) (*P* = 0.17; Table S2).

### Correlations between handgrip strength and metabolites levels

**Fig. 1:**
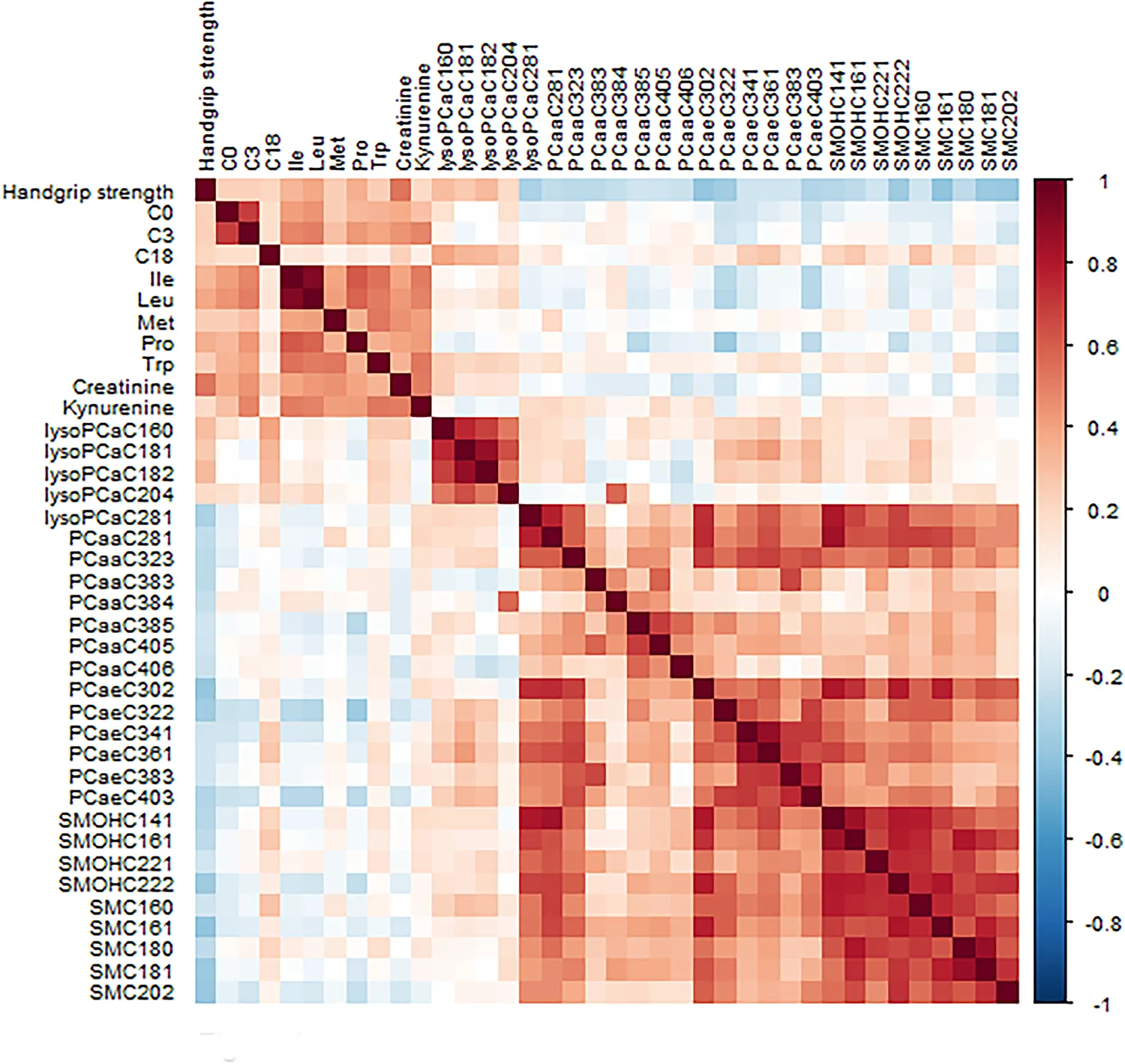
Heatmaps of Spearman correlations between baseline handgrip strength and metabolites (*q* < 0.05)

Baseline correlation analyses were performed to explore the associations between circulating metabolite levels and handgrip strength in the total study population. Of the 140 metabolites measured, 26 were significantly correlated with handgrip strength. Overall, amino acids, acylcarnitines, creatinine, and several lysophosphatidylcholines were positively correlated with handgrip strength, whereas phosphatidylcholines and sphingomyelins, including their hydroxylated forms, were inversely correlated. Correlations between baseline handgrip strength and metabolites are presented in Fig. 1.

Creatinine showed the strongest positive correlation with handgrip strength (*r* = 0.53). Among amino acids, leucine exhibited the strongest positive association (*r* = 0.38), followed by proline (*r* = 0.35), isoleucine (*r* = 0.33), tryptophan (*r* = 0.25) and methionine (*r* = 0.24). Acylcarnitines were also positively correlated, including C0 (*r* = 0.24) and C3 (*r* = 0.23). Furthermore, lysophosphatidylcholines showed positive associations, particularly lysoPCaC18:2 (*r* = 0.31), lysoPCaC16:0 (*r* = 0.30) and lysoPCaC18:1 (*r* = 0.26).

In contrast, phosphatidylcholines were inversely correlated with handgrip strength, across both PC aa and PC ae subclasses, including PCaaC38:3 (*r* = −0.26), PCaaC32:3 (*r* = −0.26), PCaeC30:2 (*r* = −0.39), and PCaeC32:2 (*r* = −0.35). Several sphingomyelins and hydroxysphingomyelins also demonstrated inverse correlations, including SMC16:1 (*r* = −0.40), SMC20:2 (*r* = −0.37), SMC18:1 (*r* = −0.36), and SMOHC22:2 (*r* = −0.36).

### Increases in handgrip strength and related metabolites

**Fig. 2:**
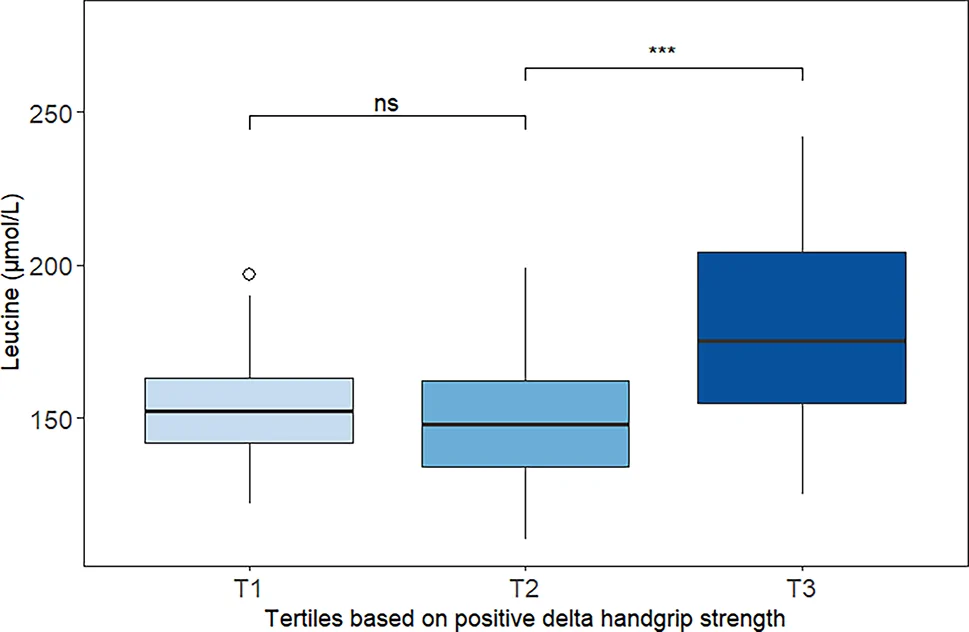
Leucine levels were compared across tertiles of positive Δ handgrip strength (ΔHGS > 0, tertiles defined by 33rd and 67th percentiles). Overall group differences were assessed using one-way ANOVA (p = 3.35 × 10⁻⁷), followed by Bonferroni-adjusted post hoc pairwise t-tests. ns, not significant, *** P < 0.001. Δ Indicates change from baseline to week 12 (post-pre). HGS, handgrip strength; T1, first tertile; T2, second tertile; T3, third tertile.

We assessed if the 26 metabolites that were significantly associated with baseline handgrip strength were also related to changes in handgrip strength (ΔHGS). Baseline levels of leucine (*r* = 0.29), isoleucine (*r* = 0.27), PCaeC322 (*r *= −0.28) and SMOHC222 (*r* = −0.26) were significantly correlated with ΔHGS improvements (ΔHGS > 0, *n* = 82). No metabolites were significantly correlated with negative changes in handgrip strength (ΔHGS ≤ 0, *n* = 89). Furthermore, in tertile analyses of ΔHGS within improvers, leucine levels increased across tertiles, with higher levels observed in individuals who achieved greater improvements in handgrip strength over the 12-weeks (Fig. 2).

### Limited impact of intervention on the metabolomic profile

**Fig. 3:**
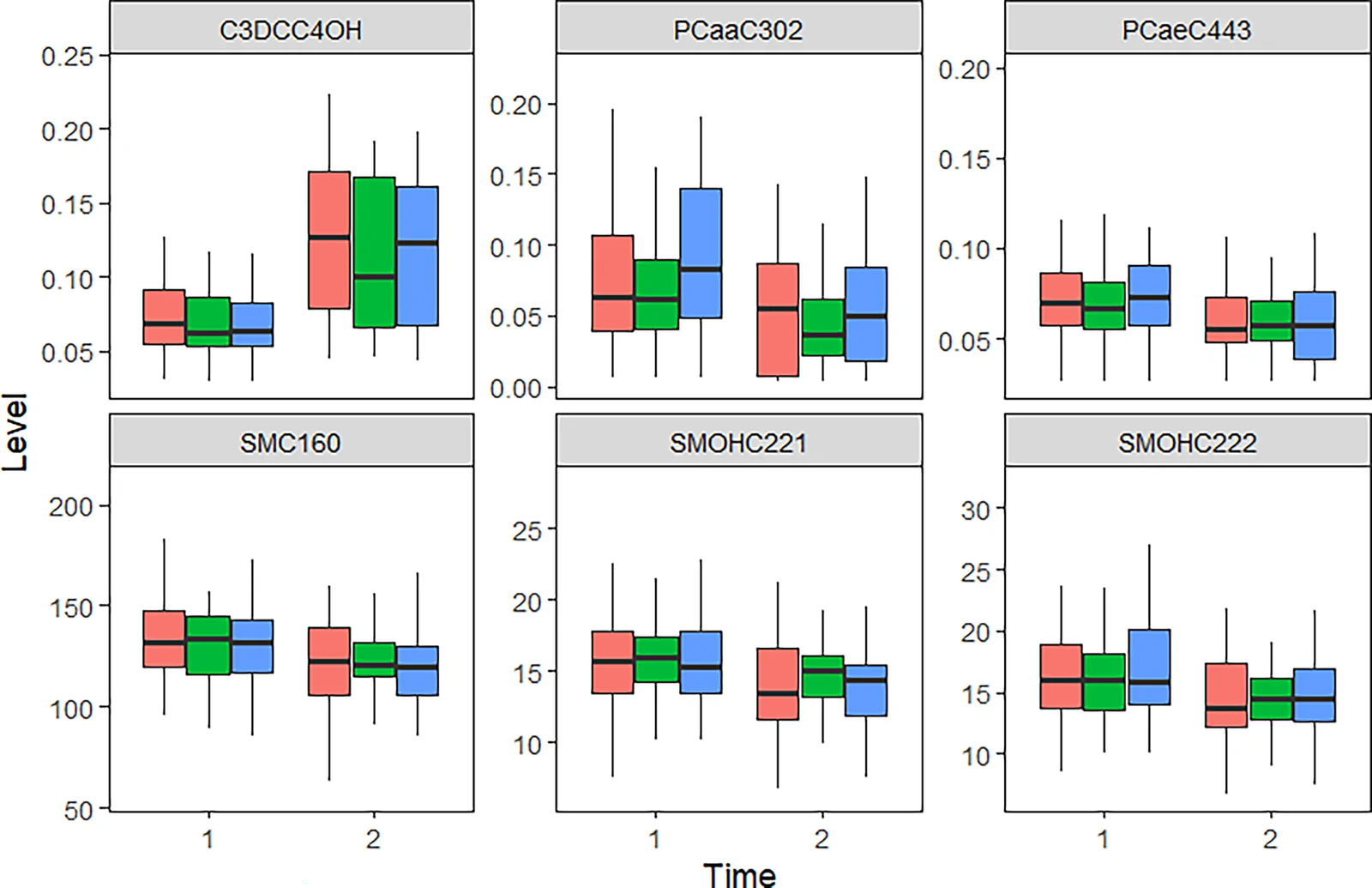
Box plots of six metabolites significantly associated with time in the ASCA analysis, shown at pre- and post-intervention for low protein (red), plant protein (green), and dairy protein (blue) groups. C3DCC4OH increased over time, whereas the remaining five metabolites decreased. No significant group or group × time effects were observed. Time 1 = pre intervention, Time 2 = post intervention

Applying ANOVA-Simultaneous Component Analysis (ASCA) to the metabolomic data identified a significant main effect of time (*p* < 0.05, using 1000 permutations) with no evidence of a group effect (*p* = 0.23) or a group × time interaction (*p* = 1.00). Six metabolites were significantly associated with the time component (C3DCC4OH, PCaaC302, SMC160, PCaeC443, SMOHC222, and SMOHC221). Across all groups, C3DCC4OH increased over time, whereas PCaaC302, SMC160, PCaeC443, SMOHC222, and SMOHC221 decreased over the 12 weeks intervention period (Fig. 3).

## Discussion

In this secondary analysis, we investigated whether a 12-week dietary consumption of a supplemental protein with various sources (plant vs. dairy) would alter circulating metabolites in healthy older adults. Overall, the metabolomic response did not differ between protein sources, with no evidence of a group effect or group × time interaction. However, metabolite profiles were associated with handgrip strength outcomes, and baseline leucine and isoleucine levels showed a positive association with improvements in handgrip strength over the intervention period.

The positive associations between handgrip strength and branched-chain amino acids (including leucine and isoleucine) are consistent with prior metabolomic studies linking higher circulating BCAAs levels to greater muscle mass and better physical performance (Lustgarten et al., 2014; Su et al., 2022). Consistent with our findings, a recent systematic review and meta-analysis reported that circulating leucine, isoleucine and tryptophan levels were significantly lower in individuals with sarcopenia compared to individuals without sarcopenia, although heterogeneity across studies was high (Dai et al., 2021). Leucine and isoleucine are essential amino acids with well-established anabolic properties, and leucine in particular is a key stimulator of muscle protein synthesis through activation of the mTOR signalling pathway (Drummond & Rasmussen, 2008), providing biological plausibility for its positive correlation with handgrip strength. The observed positive associations between baseline leucine and isoleucine with improvements in handgrip strength could suggests that, beyond total protein intake, amino acid profile, particularly leucine availability, may be relevant for functional adaptation in older adults.

In the present study, free carnitine (C0) and propionylcarnitine (C3) were positively associated with handgrip strength, which may reflect more favourable mitochondrial substrate handling and energy metabolism in older adults. Carnitine and its acyl derivatives are central to mitochondrial fatty acid transport and broader energy homeostasis (Bene et al., 2018). Together, these findings add to emerging evidence that circulating metabolites, including carnitine and acylcarnitine metabolites, may have utility as biomarkers of muscle health and physical function (Chen et al., 2024; Guo et al., 2024; Lynch et al., 2025; Seo et al., 2025). Previous work in community-dwelling older adults has linked baseline plasma acylcarnitines, particularly short-chain dicarboxylic and hydroxylated acylcarnitines, with longitudinal decline in handgrip strength (Ng et al., 2021). In men, lower circulating C5-carnitine has been reported in individuals with sarcopenia, with positive associations with skeletal muscle mass, suggesting that short-chain acylcarnitine metabolism may relate to sarcopenia-related phenotypes (Seo et al., 2025). Similarly, in patients undergoing haemodialysis, higher medium- and long-chain acylcarnitines, including C7 and C16 carnitine, were associated with stronger handgrip strength (Moorthi et al., 2024). Beyond carnitine pathways, lower handgrip strength in older adults has been linked with altered serum and faecal metabolomic profiles, particularly gut-related metabolites, and a shift in gut microbiome composition, supporting a potential gut-muscle axis in muscle health (Guo et al., 2024).

Furthermore, we observed positive associations between handgrip strength and several lysophosphatidylcholines. Notably, lysoPCaC18:2, lysoPCaC16:0 and lysoPCaC18:1 are the same species reported to be higher in individuals with greater skeletal muscle mitochondrial oxidative capacity in the Baltimore Longitudinal Study of Aging (Semba et al., 2019). These findings are consistent with recent metabolomic studies reporting lower levels of lysophosphatidylcholines in individuals with sarcopenia, with LysoPC17:0 identified as a particularly promising biomarker of sarcopenia in older adults (Han et al., 2024). Lysophosphatidylcholines are bioactive lipids generated through phospholipase A2 activity and play key roles in membrane remodelling, lipid transport, and metabolic signalling (Tan et al., 2020). Higher circulating levels in stronger individuals in this cohort may therefore reflect a metabolic state favourable to muscle maintenance, potentially through improved regulation of inflammatory processes.

Multiple phosphatidylcholine species from both PC ae and PC aa subclasses, together with sphingomyelins and hydroxysphingomyelins were inversely associated with handgrip strength. In line with our study, prior studies have reported higher circulating sphingomyelins, particularly plasma SM(16:0), in older men with lower muscle strength (Seo et al., 2024), supporting a potential role for sphingolipids dysregulation in impaired muscle function (Hannun & Obeid, 2018). Similarly, metabolomics signatures of reduced skeletal muscle mitochondrial oxidative capacity in older adults have been enriched in phosphatidylcholine and lysophosphatidylcholines, with sphingolipid metabolism, emerging as a key enriched pathway (Tian et al., 2024; Werdyani et al., 2022). Collectively, these metabolomic patterns suggest that muscle strength in ageing may reflect an interplay between anabolic amino acid metabolism and lipid remodelling. As these analyses were correlation-based, the findings should be interpreted as associative rather than causal. Prospective studies are needed to establish temporal directionality and to clarify the underlying mechanisms linking circulating metabolomic profiles with muscle strength.

There is ongoing debate regarding whether the current recommended dietary allowance for protein intake is sufficient to maintain muscle mass and function in older adults (Bauer et al., 2013; Nunes et al., 2022). Age-related anabolic resistance, reduced appetite, and alterations in protein metabolism may increase requirements for both higher total dietary protein intake and improved protein quality (Nunes et al., 2022). Evidence suggests that higher-quality, leucine-rich proteins more effectively stimulate muscle protein synthesis (Phillips, 2016). However, the functional relevance of protein source for maintaining muscle strength remains uncertain. In this cohort, despite some improvement in handgrip strength over the intervention period, higher quality protein supplementation did not significantly increase handgrip strength compared with the low-protein control. One plausible explanation is that participants were generally healthy and likely had adequate protein intake at baseline, limiting the potential for additional strength gains. In addition, participants were instructed to maintain their usual level of physical activity throughout the study, although physical activity was not monitored. Together, these factors may have reduced the likelihood of observing differential effects of the protein intervention. In line with this, a recent randomised controlled trial reported that protein supplementation in the absence of resistance exercise resulted in minimal benefits for muscle strength, particularly in non-frail population (Mertz et al., 2021). However, short-term trials show that protein can still stimulate muscle protein synthesis, for example, a randomised controlled trial showed that whey and pea protein increased myofibrillar protein synthesis in older men over a one-week intervention period (McKendry et al., 2024). Consistent with the absence of between-group differences in handgrip strength in our study population, we did not observe any significant group or group × time effects in the metabolomics data, indicating no detectable differential circulating metabolomic response by protein sources over the intervention period. However, a significant main effect of time on metabolomic profiles was observed in the absence of a group effect or a group × time interaction. This pattern points to a shared shift over the 12-week period across the groups rather than a protein source specific response. These changes may reflect broader, non-specific influences, such as study participation, modest improvements in overall dietary adequacy or other-time dependent factors (Li et al., 2022; McCambridge et al., 2014), and should be explored in future studies to isolate these effects. Furthermore, it is important to acknowledge that a broader and more comprehensive metabolomic profile may have detected changes in metabolites not present in our platform.

This study used a targeted metabolic profiling combined with both cross-sectional and longitudinal analyses, enabling characterisation of metabolic responses over time and between groups. The repeated-measures design supports detection of within-participant change and reduces confounding from inter-individual variability. Moreover, the application of ASCA provided a robust multivariate framework to disentangle time- and group-related effects, improving interpretation of complex, correlated metabolite patterns. However, limitations include the sample size, which may reduce statistical power and limit generalisability. Furthermore, as findings are based on circulating metabolites, they may not fully capture intramuscular metabolic processes, and tissue-specific adaptations may therefore be underrepresented. In conclusion, metabolomic responses were similar across protein types. However, circulating metabolite levels were associated with handgrip strength outcomes, and these findings should be validated in future intervention studies.

## Supplementary Information

Below is the link to the electronic supplementary material.

Supplementary Tables S1-S2.

## Data Availability

The data are available upon reasonable request.

## Abbreviations

BCAAs: Branched-chain amino acids
FDR: False discovery rate
HGS: Handgrip strength
PC: Phosphatidylcholine
SM: Sphingomyelin
