# Supplementary material for: Association of handgrip strength with the plasma metabolomic profile: secondary analysis of a protein intervention study
Source: Metabolomics. 2026 Jun 30;22(4):106. doi: 10.1007/s11306-026-02496-4 (PMC13319504; doi:10.1007/s11306-026-02496-4)
Supplement: Supplementary file 1 — Supplementary Tables S1-S2. [file 11306_2026_2496_MOESM1_ESM.docx]

**Table S1.** Baseline characteristics of participants included in the secondary analyses.

| **Variable** | **Overall**  **n=171**^1^ | **Low Protein**  **n=55**^1^ | **Plant Protein**  **n=60**^1^ | **Dairy Protein**  **n=56**^1^ | **P-Value**^2^ |
| --- | --- | --- | --- | --- | --- |
| Age, years | 59.2 (7.7) | 60.2 (8.6) | 59.0 (7.4) | 58.5 (7.3) | 0.520 |
| Female/Male, n | 100/71 | 28/27 | 36/24 | 36/20 | 0.348 |
| BMI, kg/m^2^ | 26.2 (4.9) | 26.6 (4.8) | 25.8 (4.8) | 26.3 (5.3) | 0.689 |

^1^Values are presented as mean (SD) for continuous variables. ^2^ P values were calculated using one-way ANOVA for continuous variables and Pearson’s χ² test for categorical variables. Full baseline characteristics have been published previously (30). BMI, body mass index.

**Table S2.** Handgrip strength at baseline, post-intervention and changes (Δ).

| **Variable** | **Overall**  **n=171**^1^ | **Low Protein**  **n=55**^1^ | **Plant Protein**  **n=60**^1^ | **Dairy Protein**  **n=56**^1^ | **P-Value**^2^ |
| --- | --- | --- | --- | --- | --- |
| Baseline HGS, kg | 27.9 (9.0) | 28.7 (10.1) | 27.1 (8.4) | 28.0 (9.0) | 0.609 |
| 12-weeks, HGS, kg | 28.4 (9.0) | 29.6 (10.0) | 27.7 (8.6) | 27.9 (8.5) | 0.472 |
| Δ HGS, kg | 0.5 (3.0) | 0.9 (3.4) | 0.6 (3.2) | -0.1 (2.4) | 0.165 |

^1^ Values are presented as mean (SD).^2^ P values were calculated using one-way ANOVA for baseline and post-intervention and Welch’s one-way ANOVA for delta (Δ) handgrip strength. Δ Indicates change from baseline to week 12 (post-intervention - baseline). HGS, handgrip strength.
